# Modulation of quinpirole-induced compulsive-like behavior in rats by environmental changes: Implications for OCD rituals and for exploration and navigation

**DOI:** 10.1186/1471-2202-8-23

**Published:** 2007-03-26

**Authors:** Pazit Zadicario, Sharon Ronen, David Eilam

**Affiliations:** 1 Department of Zoology, Tel-Aviv University, Ramat-Aviv 69978, Israel

## Abstract

**Background:**

Rats treated chronically with the D_2–3 _dopamine agonist quinpirole were previously proposed as an animal model of obsessive-compulsive disorder (OCD) since their behavior is based on repeated, compulsive-like persistent traveling between a few places in the open field. The aim of the present study was to determine properties of the physical environment that shape such behavior. For this, quinpirole-treated rats were first exposed to an arena with an array of objects (landmarks) and after the development of compulsive-like behavior, the arena was manipulated by multiplying the number of objects, changing their spacing, relocating object array, or removing the objects.

**Results:**

When the number of objects was retained but they were spaced further apart, rat routes converged at 1–2 of the objects and at the corner at which the rats had been introduced into the arena (start corner). When object spacing was retained but their number was increased, the rats traveled between the objects with the routes converging only at the start corner. Finally, when object array was relocated to different places within the arena, the rats extended their routes from the start corner to the object array, regardless of array location.

**Conclusion:**

Quinpirole-treated rats organized and updated their progression primarily according to the proximal layout of landmarks, but did so with excessive repetitions compared with saline-treated rats. The behavior of quinpirole-treated rats paralleled human OCD rituals that are linked to the immediate physical environment, featuring an excessive rate of performance. Finally, when only a few objects were present, they were perceived by the rats as positional cues (beacons) that routes converged at them. In contrast, in the presence of many objects, the routes passed between the objects as if using them as directional cues.

## Background

To navigate in the world, animals use various cues [1-3]. Objects or landmarks constitute external cues in the layout ('landscape') of the environment, in reference to which an animal can locate itself [4,5]. In general, motor behavior has a strong affinity with specific locations in the environment [6], and animals perceive the location of objects and use this information in exploration and navigation in the environment. For example, salient landmarks associated with specific locations function to help animals in returning to particular locations [1], and navigation in rats appears to be primarily based on the geometric arrangement of landmarks [7]. In the present study we manipulated an array of landmarks in order to examine how motor behavior in rats is affected by the number of objects, their density, their spacing and their location in the test environment. Specifically, we asked the question: What in the environment makes rats repeatedly travel to a specific object? Do the rats travel to a specific object or to a specific location? If traveling to a specific location, how is that location determined: in relation to the global room frame or in relation to some local cues?

The above questions were posed not only in the context of exploration and navigation of normal rats, but also in rats treated with the psychoactive drug quinpirole – a dopamine D_2_/D_3 _receptor agonist [8-10]. We chose to study navigation and exploration in rats under quinpirole for two reasons: 1) their repetitive locomotion; and 2) their relevance for the study of obsessive-compulsive disorder (OCD). Although quinpirole-treated rats may freely move in a given area, they repeatedly travel between only a few locations [11,12]. We presumed that such repetitive behavior would unambiguously illuminate how environmental cues shape exploration and navigation – a target of the present study. Specifically, rats treated chronically with quinpirole ceaselessly move between the same few objects/locations, and their locomotion is therefore strongly coupled with the environment. After 10 repeated quinpirole injections (0.5 mg/kg per injection, with injections spaced at 3–4 day intervals) rats were sensitized to the drug, reaching a level of activity that could be as much as 16-fold higher than in controls. This elevated level of activity, however, was confined to a restricted portion of the arena, within which the rats locomoted hurriedly between a few objects/locations, seemingly exploring the environment with unbounded curiosity without habituation or fatigue [11]. Repetitive traveling between the same few objects raises the question of whether the rats travel from one specific object (landmark) to the next, or between specific locations in the environment. The former possibility was tested by moving objects apart, and by adding or removing objects. The latter possibility was tested by relocating the arena to different locations within the test room. Together, these tests were expected to reveal how environmental cues shape this behavior, and what is the contribution of the various facets of an object that make it a target in traveling: its location within the arena or in the room, its distance from other objects, or its distinctness against the background. These facets can be easily discerned in quinpirole-treated rats and compared with saline-treated rats in order to extend the results to exploration and navigation in general.

A second reason to study quinpirole-treated rats was that their behavior is considered a model of OCD [13], in meeting a set of ethologically-derived criteria of compulsive behavior [12]. These criteria were recently applied in studying rituals in OCD human patients [6], revealing that, as in quinpirole-treated rats, compulsive rituals in OCD patients are composed of relatively few motor acts that are organized in a flexible yet recurrent manner. Moreover, rituals in OCD patients feature a strong affinity to a few specific locations or objects [6,14], as also shown in quinpirole-treated rats [12,15]. This parallel between OCD patients and quinpirole-injected rats raised the question of what are the physical properties that account for the coupling of motor rituals with specific objects/locations. Identifying such properties was the target of the present study, and for this we tested how rats respond to environmental manipulations. Specifically, rats were expected to change their behavior following a change in object layout through modifying the number of objects, their spacing, density, or location. On a broader level, we asked how the spatiotemporal structure of locomotor behavior is modulated by objects in the immediate vicinity of the animal, and whether compulsive motor rituals are resistant to changes in the layout of local environmental landmarks.

### Experiment 1: Rituals and attraction to objects: the effect of the number of objects and inter-object distance under 'elastic' and 'symmetrical' environment enlargement

#### Experimental design and rationale

Quinpirole-treated rats ceaselessly move between the same few objects/locations, and their locomotion is therefore strongly coupled with the environment. One possibility is that they move from object to object regardless of distance (spacing) between the objects. If so, spacing the objects further apart will not modify the tendency to travel between the objects – that is, piloting between local cues laid in a fixed geometric array. Another possibility, however, is that rats pilot between locations, regardless of the presence of an object in these locations. This implies that the rats are traveling fixed distances, and if an object is spaced further apart, the rats will not reach the new object location but keep traveling to the former location, relying on global cues. Finally, quinpirole-treated rats tend to select only 1–2 objects and move between them while ignoring the other objects [12]. Consequently, we asked why certain objects were selected as the goal in traveling.

The starting point of this set of experiments was at injections 9–10, when the rats were still being injected in the small (1 × 1 m) arena with four equispaced objects. At subsequent injections 11–16 the rats were tested in one of the following environmental settings:

(i) *Elastic enlargement*:- Large arena (2 × 2 m) with four equispaced objects. This setting reflected an "elastic" enlargement of the small arena in which rats had been tested in injections 1–10.

(ii) *Symmetrical enlargement*:- Large arena (2 × 2 m) with 16 objects. This setting reflected a constant object density with both area and object number being four-fold greater, as if the small arena had simply been multiplied.

(iii) *Fixed area*:- Small arena (1 × 1 m) with four equispaced objects as in injections 1–10. This provided a reference for the above enlargements.

**Figure 1:**
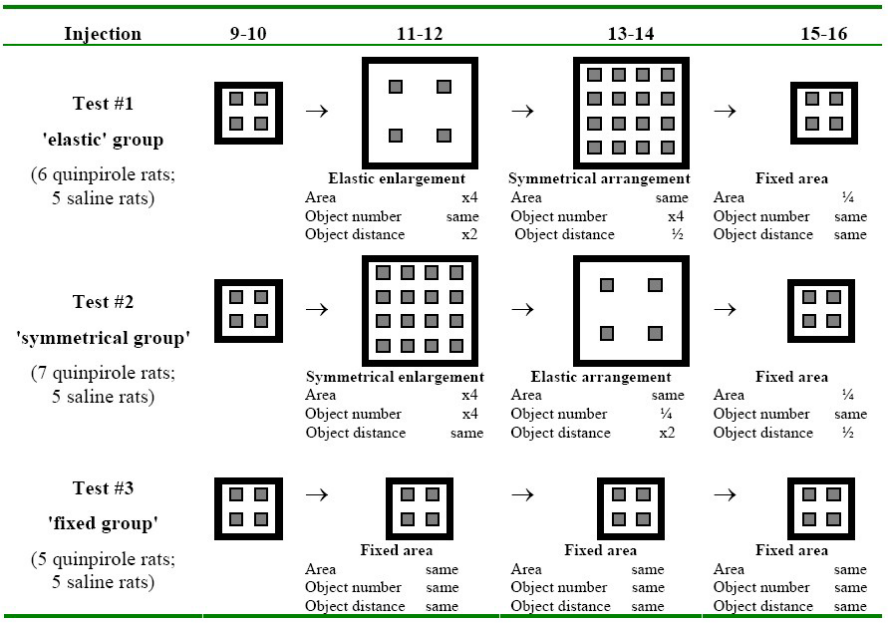
Experimental design of elastic and symmetrical arena expansion. The properties of each change in environmental settings are outlined, compared with the starting (injections 9–10) and final (injections 15–16) settings.

Rats were divided into the above three test groups, each comprising: i) a subgroup of 5–7 rats treated with 0.5 mg/kg quinpirole; and ii) another subgroup of 5 rats administered with equivolume of saline. Test group #1 ('elastic' group) was tested first under elastic enlargement (injections 11–12), then under symmetrical enlargement (injections 13–14), and finally retested in the small arena (injections 15–16) where it had been tested in injections 1–10 (Figure 1, top row). Test group #2 ('symmetrical' group) was tested in a reciprocal order (Figure 1, center row), and test group #3 ('fixed' group) was tested in the same small environment throughout (Figure 1, bottom row). Since previous studies had revealed higher variability in quinpirole-treated rats [11], these groups had a greater number of rats than the control.

The rationale for this design was as follows: Test # 1 ('elastic' group) was designed to reveal whether rats travel between objects regardless of inter-object distance or the distance of the objects from arena walls. If the rats are traveling between objects, then the number of stops at objects and route shape will not be affected by this enlargement. However, if the rats travel from object to object without stopping, then interstop distance is expected to increase. Alternatively, they may stop on the way to the now more distant objects, in which case the total number of stops will increase. Test #2 ('symmetrical' group) was designed to reveal whether the rats are equally attracted to all objects. If so, then when the arena is symmetrically enlarged, rats will travel between more objects compared to their travel on arena with fewer objects. A comparison of groups # 1 and # 2 was designed to highlight the effect of testing order. Retesting in the small arena was designed to uncover whether behavioral changes were reversible, and test group # 3 ('fixed' group) provided a control for the above comparisons. Saline groups were tested to distinguish effects that were specific to quinpirole.

### Results of Experiment 1

#### Traveled Distance

**Figure 2:**
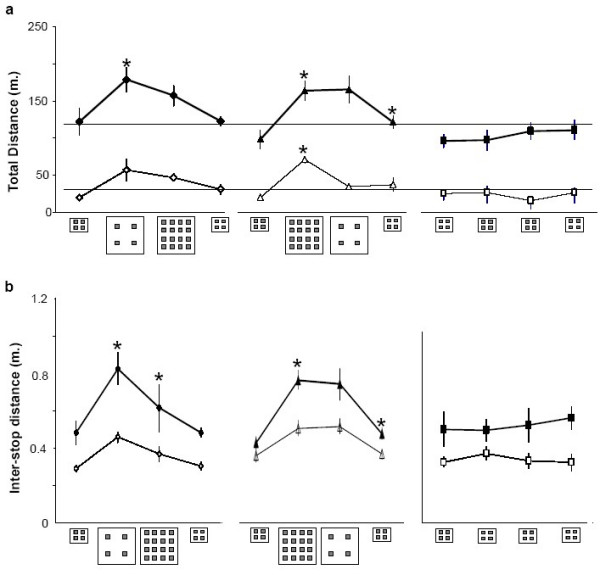
Total distance (a) and interstop distances (b) traveled by quinpirole (dark symbols; thick line) and saline (clear symbols; thin line) rats in each of the experimental groups (means ± SEM, in meters). Environmental settings are depicted along the x-axis for each group. * indicates a significant difference compared with the previous test setting (previous data point on the graph).

Quinpirole-treated rats traveled greater distances compared with saline-treated rats in the three test groups: the 'elastic' group (Two way ANOVA, between-group factor, F_1,9 _= 38.9; p < 0.001; Figure 2a, left), the 'symmetrical' group (Two way ANOVA, between-group factor, F_1,10 _= 30.3; p < 0.001; Figure 2a, center), the 'fixed' group (F_1,8 _= 35.2; p < 0.001; Figure 2a, right). Thus, quinpirole-treated rats, regardless of the setting and environmental changes, traveled greater distances compared with their saline-treated controls.

There were also differences between rats tested first under elastic and then symmetrical enlargement ('elastic' group; within-group factor, F_3,27 _= 6.3; p = 0.002; Figure 2a, left), and in rats tested first under symmetrical and then elastic enlargement ('symmetrical' group; F_3,30 _= 16.3; p < 0.001; Figure 2a, center). Indeed, traveled distance increased when rats were first introduced to an enlarged arena, either with four or 16 objects (Figure 2a). When rats in both these groups were retested in the setting of a small arena with four objects, traveled distance reverted to the initial level that these groups had displayed before the changes in environmental setting. This final level was identical with the final level in an environmental setting that was not modified ('fixed group'; Figure 2a, right – see the horizontal dotted reference line). Rats in the 'fixed group' preserved traveled distance at a constant level, similar to that observed when the other groups were tested in the same setting of a small arena with four objects. Thus, there was a typical level of activity in the small arena, which was not affected by previous exposures to a large arena.

The interaction between treatment (quinpirole *vs*. saline) and changes in environmental setting was not significant in all three test groups: i) in rats that were exposed first to elastic and then symmetrical enlargement ('elastic' group; F_3,27 _= 0.5; ns); ii) in rats that were exposed first to symmetrical and then elastic enlargement ('symmetrical' group; F_3,30 _= 3.6 ; ns); and iii) in rats that were repeatedly exposed to the same setting ('fixed' group; F_3,24 _= 0.6; ns). This constancy confirmed the similar effect of environmental changes on both quinpirole and saline groups (Figure 2a). However, the effect was faint in the less active, saline-treated rats compared with the hyperactive quinpirole-treated rats. In all, the increase in traveled distance was area-dependent, reversible, and independent of the number of objects.

#### Inter-stop Distance

Rats of the 'elastic' group significantly changed interstop distance over the various test settings (within-group factor in two-way ANOVA; F_3,27 _= 18.5; P < 0.001). Specifically, they increased interstop distance when introduced into a large arena with four objects spaced at a greater distance than in a small arena; and subsequently, when tested in a large arena with symmetrical object setting and shorter distance between objects, they reduced interstop distance. Thus, interstop distance seemed to be adjusted to inter-object distance (Figure 2b, left), as hypothesized above in the design of this test.

Rats in the 'symmetrical' group also changed interstop distance over the various test settings (within-group factor in two-way ANOVA; F_3,30 _= 26.2; P < 0.001). Specifically, they increased interstop distance when introduced into the large arena in which there were more objects spaced at a similar distance as that in the small arena. Subsequently, they preserved the same interstop distance when tested in the large arena with four-object setting and greater distance between objects. Thus, these rats seem to adjust interstop distance to arena size and not to inter-object distance (Figure 2b, center). There was no change in controls tested throughout in the small arena (Figure 2b, right).

The interaction between treatment (quinpirole *vs*. saline) and changes in environmental setting was not significant in all three test groups, confirming a similar effect of setting changes on both quinpirole and saline groups (Figure 2b). However, the effect was faint in the less active saline-treated rats compared with the hyperactive quinpirole-treated rats. In all, interstop distance seemed to be adjusted to inter-object distance in rats tested first under 'elastic' enlargement, and to arena size in the rats tested first under 'symmetrical' enlargement.

**Figure 3:**
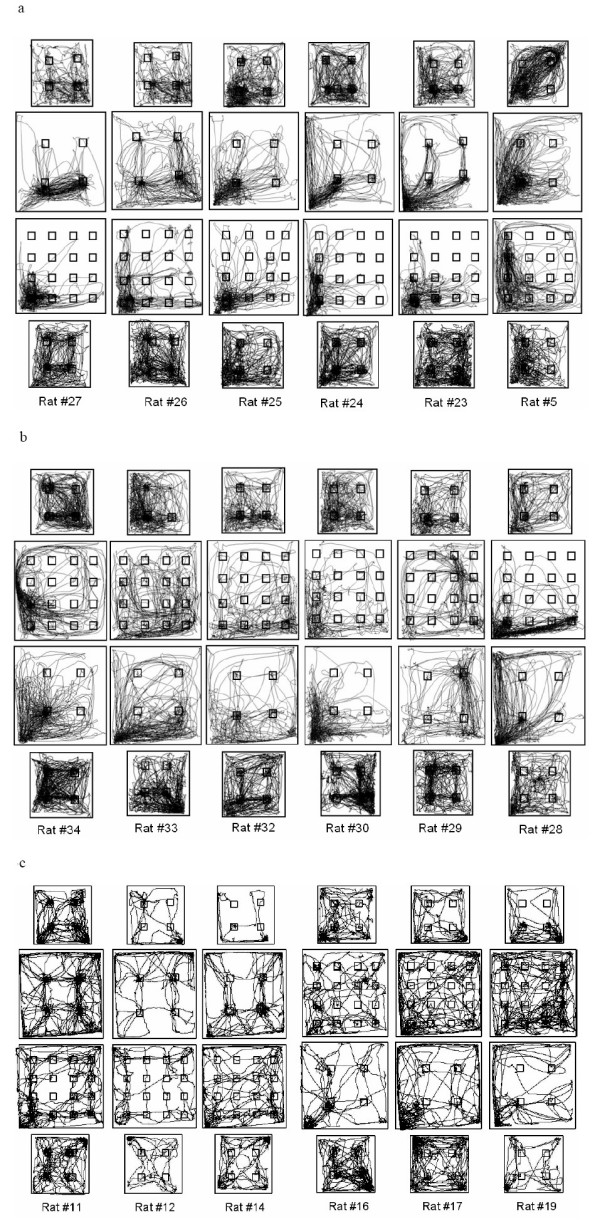
Routes of progression are shown for six quinpirole-treated rats that first underwent elastic, and then symmetrical enlargement (a), six quinpirole-treated rats that first underwent symmetrical and then elastic enlargement (b), and six saline-treated rats (c) of which three underwent the procedure shown in a) and another three underwent the procedure shown in b). Each column depicts four sessions of the same individual rat under each test condition. (Note that each rat was tested twice in each of these settings, of which only one is depicted above). It should be noted that the overall number of rats in this test was limited by the need to inject all animals twice a week in the same arena and within the same testing hours and light phase.

The difference between the behavior under 'symmetrical' and 'elastic' enlargement is shown in Figure 3. When quinpirole-treated rats first underwent elastic enlargment, their routes converged upon the objects and/or the left-bottom "start" corner, where the rats had been initially introduced into the arena (Figure 3a). The longer distance between objects resulted in a higher interstop distance (Figure 2b). When then introduced into the symmetrically enlarged arena, their routes still converged upon some of the objects at left-bottom area. The shorter distance among objects and to the left bottom corner in this setting compared with the previous elastic setting could now account for the shorter interstop distance (Figure 2b). When quinpirole-treated rats first experienced the symmetrical enlargement (Figure 3b), they displayed relatively long routes that did not converge upon objects, but passed in between them and typically converged only upon the starting corner. The same structure of routes was preserved when these rats were then introduced into the elastic setting. Since the routes in these rats typically did not converge upon specific objects, interstop distance was not affected by these changes, but adjusted solely to arena size (Figure 2b). Figure 3c depicts the routes of saline-treated rats. Despite their lesser activity, it is notable that routes converged upon objects in saline-treated rats tested first under elastic enlargment, whereas those that were first tested under symmetrical enlargment traveled more between objects (Figure 3c).

**Figure 4:**
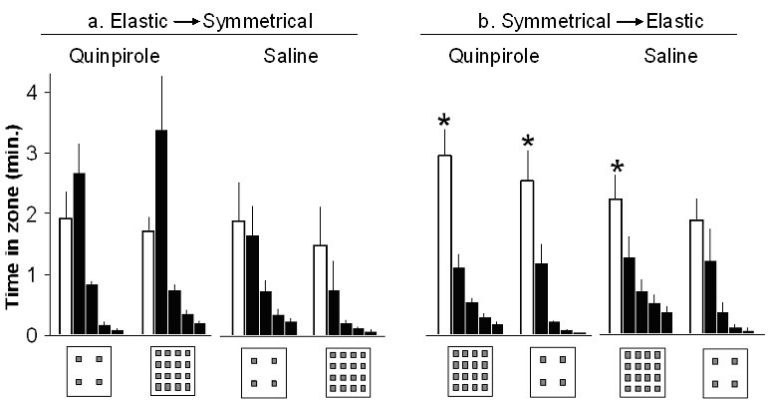
Stay duration (means ± SEM, in minutes) in quinpirole-treated and in saline-treated rats exposed first to elastic and then to symmetrical object-setting (a) and rats exposed first to symmetrical and then to elastic object-setting (b). For each group five times (bars) are shown: the time spent in the spaces between objects (clear bar) and the time at four objects with the longest duration (dark bars; ranked from left to right in decreasing order). * indicates a significant difference between the time spent between the objects (clear bar) and the first-ranked object in each inset (the left dark bar in each inset) as revealed in a Paired t-test comparison in each test group. As shown, in both saline and quinpirole-treated rats that were exposed first to elastic and then to symmetrical setting (a), the time spent at the first-ranked object (the leftmost dark bar) was not significantly different from the time spent at the spaces between objects (clear bar). In contrast, rats that were first exposed to symmetrical and then elastic setting (b) consistently spent more time at the spaces between the objects compared with the first-ranked place (t_13 _= 3.5; p = 0.03 in quinpirole; t_9 _= 3.2; p = 0.01 in saline), indicating that these rats traveled more in the spaces between the stones rather that staying at/on some of the objects, as did rats that were tested in the large arena with four objects (elastic groups). This form of traveling in the space between objects was preserved when the number of objects was then reduced form 16 to four, but this time the difference between traveling in the spaces between objects and the first-ranked object was significant only in quinpirole-treated rats (t_13 _= 3.3; p = 0.01).

Figure 4 depicts the difference between rats that were first exposed to elastic enlargment and apparently traveled from object to object, and those that were first exposed to symmetrical enlargement and traveled in the spaces between the objects. As shown, exposure to a large arena with only four objects resulted in spending extended periods at one or two of the objects, with the time spent between objects not being significantly different from that spent at the most visited object. In contrast, in quinpirole- and saline-injected rats that were exposed to a large arena with 16 objects, time spent in the spaces between objects was significantly longer than time spent at the most visited object.

### Experiment 2: Location in reference to arena walls or room setting: 'four-wall' shift or 'two-wall' shift, followed (respectively) by 'translocation' or 'removal' of objects

#### Experimental design and rationale

The present set of experiments was designed to evaluate how the location of objects in reference to room frame and their distance from the arena walls might affect the spatiotemporal structure of locomotor behavior. For this we preserved the distance between objects, keeping the same array of four objects and spacing as in the first 10 injections, but manipulated the location of the array within the arena, or the location of the arena within the room. The question posed here was whether rituals are performed at specific locations in the global environment, or whether they are coupled with specific objects regardless of their location in the near environment.

**Figure 5:**
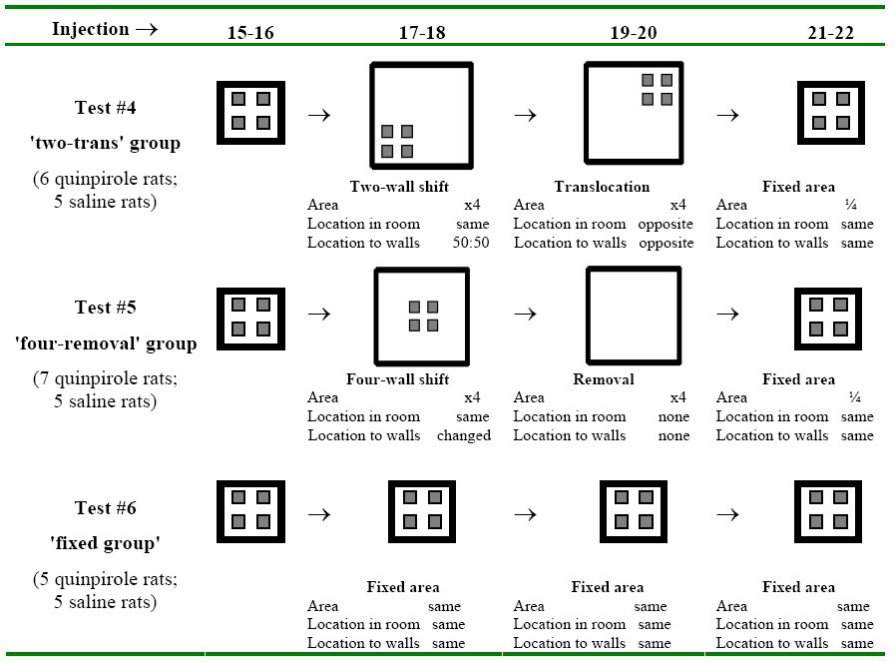
Experimental design of wall shifting and object translocation or removal. The properties of each change in environmental settings are outlined, compared with the starting (injections 15–16) and final (injections 21–22) settings. Saline-treated rats were tested, as indicated for each test group, but their data are not shown since they were hypoactive.

Saline-injected rats had become entirely habituated to the environment at this stage of testing (16 successive exposures to the open field). They hardly locomoted, and virtually did not respond to changes in environmental settings. Including the saline-injected groups in statistical comparisons could therefore result in significant differences compared with quinpirole-injected rats, but these differences would be meaningless considering the rats' dull behavior under saline. Therefore, although saline-treated rats were tested and analyzed, the following analyses refer only to quinpirole-treated rats. Behavior of these rats was analyzed to reveal whether they traveled in relation to object location (proximal cues), in relation to arena walls, or to room setting (distal cues). It is noteworthy that quinpirole-injected rats can be challenged over a long period of chronic injections: after the first 10 injections, they preserve their behavioral patterns as long as the chronic injection regime continues, up to 40 injections and even more [11]. To confirm here that they indeed preserve their behavioral patterns, rats were retested in the original 1 × 1 m arena at the beginning and end of this set of experiments. These experiments started with injections 15–16, when rats were injected in the small arena (1 × 1 m) with four equispaced objects. At injections 17–20, rats were tested in one of the following environmental settings:

(i) *Two-wall shift*:- In switching from a small (1 × 1 m) to a large arena (2 × 2 m) the four equispaced objects remained in the same location near the starting corner, as if two arena walls had been moved further away from the object array, and the objects remained in the same location in the room and in relation to two of the arena walls (Figure 5).

(ii) *Four-wall shift*:- In switching from a small (1 × 1 m) to a large arena (2 × 2 m) the four equispaced objects remained in the same location in relation to the room frame, as if the four walls had been moved further away from the object array while the array remained in the same location in the room, but now located further away from the arena walls (Figure 5).

(iii) *Translocation*:- the four-object array was moved to the opposite arena corner.

(iv) *Removal*:- the four objects were removed from the arena.

(v) *Fixed area*:- small arena (1 × 1 m) with equispaced four objects as at injections 15–16.

Rats that were injected with quinpirole at injections 1–16, were re-divided into three test groups, balanced for the previous groups of the previous experiment, and arranged in experimental groups as shown in Figure 5.

The rationale of this design was to distinguish whether quinpirole-injected rats were moving in reference to room setting (= a specific region of the room), in relation to arena walls (= a specific region of the arena), or in relation to the object array regardless of its location in the room or in the arena. In Test #4, the object array remained in the same sector in relation to room setting and start corner, but we moved two walls further away. If the rats were just traveling from the start corner to the object array, then the enlargement should not change their behavior, indicating that the increased activity in Experiment 1 was due to object relocation and not due to arena enlargement. In the subsequent phase, rats were challenged with translocation of the object array to the opposite corner. If behavior is solely dictated by the object array regardless of its location in the room and the arena, rats should transfer their activity to the new location of the array. However, if they also organize their behavior in relation to the room and/or arena, they should stretch their routes to the new location of the object array when this is relocated at the opposite corner. In Test group # 5, the same subject matter was addressed. In the four-wall shift, we tested whether the rats were moving in a certain sector of the room (the object array), or also in relation to the arena walls that were now further apart. Finally, the objects were removed to highlight their effect. At the end of this set of experiments, all rats were re-tested in the small arena (injections 21–22) to determine whether behavioral changes were reversible. Test group # 6 ('fixed' group) provided control for the above comparisons.

### Results of Experiment 2

#### Four-wall shift and two-wall shift

**Figure 6:**
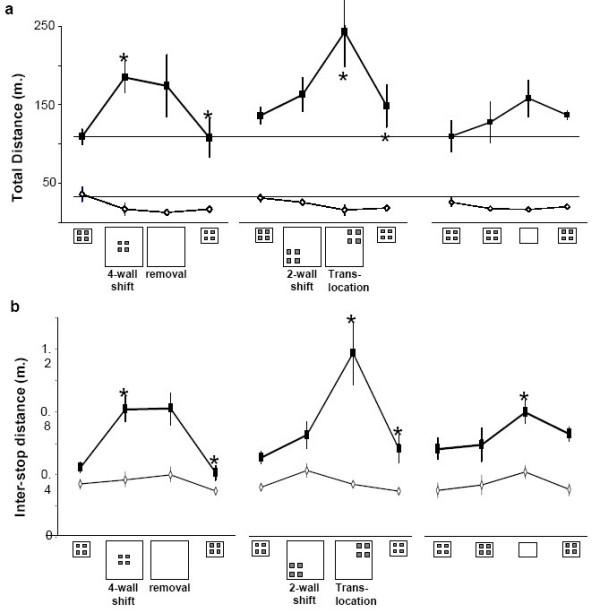
Total distance (a) and interstop distances (b) traveled by quinpirole (dark symbols; thick line) and saline (clear symbols; thin line) treated rats in each of the experimental groups (means ± SEM, in meters). Environmental settings are depicted along the x-axis for each group.

**Figure 7:**
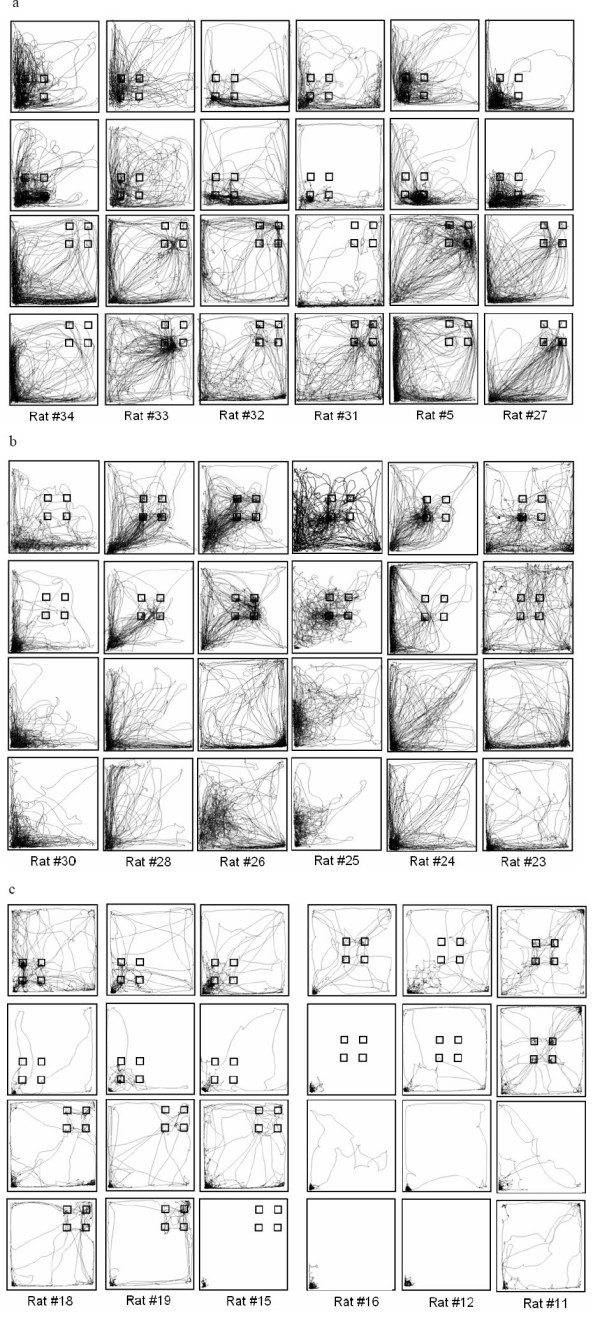
Routes of progression are shown for six quinpirole-treated rats that first underwent two-wall shift, and then translocation (a), six quinpirole-treated rats that first underwent four-wall shift and then removal (b), and six saline-treated rats (c) of which three rats underwent the procedure shown in a) and another three rats underwent the procedure shown in b). Each column depicts routes of progression in one individual rat during the two session under one test setting, and then two sessions under the subsequent test session. (Note that each rat was tested twice in each setting, of which both are depicted above).

Traveled distance significantly increased when the object array remained in the center while the four arena walls shifted away (within-group ANOVA; F_3,30_= 8.7; P < 0.001; Figure 6a, left), but not when the object array remained in the same location and distance to the start (left bottom) corner with only two walls shifted away (within-group ANOVA; F_3,27 _= 6.3; P = 0.002; Figure 6a, center). There was no significant change in the group injected in a fixed arena structure (within-group ANOVA; F_3,24 _= 1.1; P = ns; Figure 6, right). As shown in the trajectories of locomotion of these rats (Figure 7a), they extended their routes from the bottom/left walls to the center, where the object array remained under four-wall shift, but when the object array remained close to the bottom/left walls under two-wall shift, the rats remained in the same area, virtually ignoring the new space added (Figure 7b). Together, responses to these changes demonstrate that the rats were moving from the start corner to the object array, regardless of its location in the arena or in the room. Consequently, in two-wall shift when the object array remained near the start corner, the rats virtually ignore the added space of the enlarged arena (Figure 7).

#### Removal and Translocation

When the object array was translocated compared with its location under the two-wall shift, the rats profoundly increased traveled distance (Figure 6a, center). This is illustrated in the trajectories of locomotion: these rats remained in a confined sector of the arena when the objects were located near the start corner, extended their routes to the center of the arena when the objects were placed there, and further extended their routes to the further translocated objects (Figure 7). This result reconfirms the above finding that the rats were traveling from the start corner to the array regardless of its location.

When the array was removed, the rats preserved the greater traveled distance that they had displayed under four-wall shift (Figure 6a, left). Their routes in the removal test (empty arena) demonstrate meandering in the center without converging upon any specific location except for the start corner. To some extent, locomotion routes in these rats followed the arena walls, which in the lack of objects were the salient physical structure (Figure 7). Similar effect was obtained when the objects were removed from the small arena in injection 21 (Figure 6a,b; data not shown).

**Figure 8:**
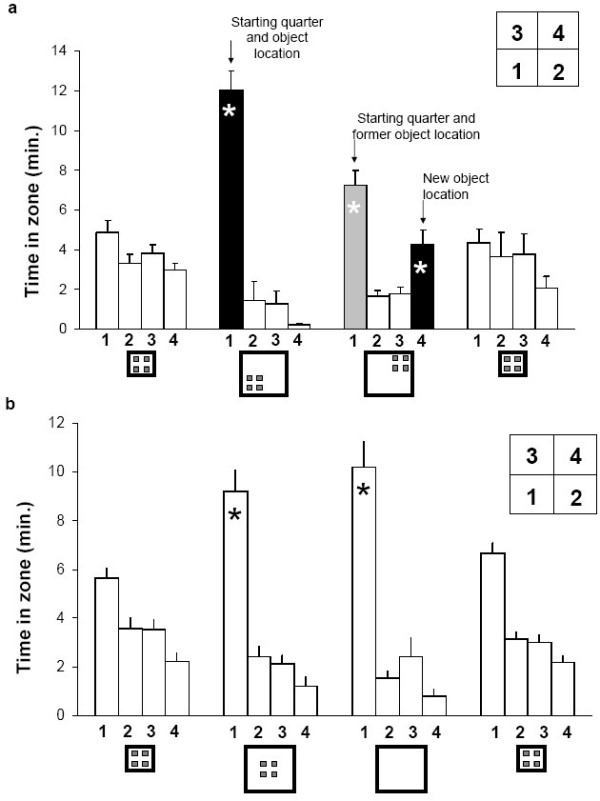
Stay duration (means ± SEM, in minutes) in each quadrant of the arena, in the groups that first underwent two-wall shift and then translocation (a) and the groups that first underwent four-wall shift and then removal (b). Environmental setting is depicted at the x-axis and quadrants labeling at the top left. In two-wall shift, rats remained in quadrant 1 (which is equal to the entire arena before expansion). When objects were then translocated, the rats split their time between the starting quadrant (1) and the quadrant that now contained the object array (4). In four-wall shift the rats spent most of the time in quadrant 1, which contained both the start corner and the bottom left object. This pattern was not affected when the objects were then removed, indicating that the rats kept traveling in the same quadrant (see Figure 7).

Interstop distance in this set of environmental changes (Figure 6b) corresponded to the changes in total traveled distance (Figure 6a), reconfirming the above finding that the rats were traveling from the start corner to the object array (within-group ANOVA; F_3,30 _= 17.9; P = 0.0003 for '*four-wall shift*'; F_3,27 _= 14.1; P < 0.001 for '*two-wall shift*'; F_3,24 _= 3.2; P = ns for 'fixed arena'). In other words, the further the objects were located in relation to the start corner, the greater were the overall traveled distance and inter-stop distance, with an insignificant effect of arena size. An illustration of the effect of array location on locomotor activity is shown in Figure 8.

## Discussion

### Summary of results

1. Rats were first accustomized to a specific environment and then introduced to changes in the landscape in order to evaluate how they re-adjusted to the new environmental setting.

2. As long as the environment setting remained unmodified, the rats displayed the same level of activity and the same routes of progression.

3. When the arena was expanded and object spacing was modified ('elastic' enlargement), rats mostly traveled from the start corner to 1–2 of the objects, and preserved this pattern even after a subsequent addition of objects. Thus, these rats were moving between specific objects regardless of their distance or location in the local environment.

4. In contrast, when rats were exposed to a large arena with a higher number of equispaced objects ('symmetrical' enlargement), they took roundtrips to the start corner, crossing the spaces between the objects. These rats were not traveling to specific objects or locations, and did not alter their behavior under subsequent object removal.

5. Quinpirole-injected rats moved between the start corner and an object array regardless of array location; the further the array the longer the distances they traveled. This implies that the rats were not moving to a specific location in the global room panorama, but rather traveling from the start corner to a local set of cues regardless of their location

6. When object array remained near the start corner or when removed from the arena, quinpirole-treated rats remained in the vicinity of the start corner, as if ignoring the added arena space. This implies that the increased activity in an enlarged arena with relocated objects was mainly a result of object relocation.

7. Thus, rat routes were primarily anchored to the start corner and from there extended to the object array (if present) regardless of the array location.

### A hypothesized mechanism of ritual formation may explain the response of quinpirole-injected rats to arena enlargement

Persistence of repetitive behavior under quinpirole is considered as an model of human OCD, either in animals tested in a T-maze [16-18] or in freely-moving animals in an open field [12]. Indeed, behavior of quinpirole-injected rats has the characteristics of compulsive checking, thus providing a pharmacology-derived model of OCD. The characteristics derived from observing motor rituals in rats have been applied in studying motor rituals in OCD patients [6]. In a recent account it was hypothesized that rituals develop by shifting focus in action parsing, from mid-ranged actions to finer movements [19]. According to this hypothesis, behavior is generally categorized at three levels: i) simple gestures; ii) behavioral episodes; and iii) scripts. An excessive focus on the level of simple gestures is what happens in cultural and individual rituals (for example, in OCD), in contrast to spontaneous focusing on the mid-ranged behavioral episodes in normal behavior [19-21]. In the context of rats in the open field, we suggest that the basic level of simple gestures is that of stops (visits) at specific locations. The mid-range level of behavioral episodes is that of roundtrips from the start corner (or the home base). This mid-range level of roundtrips is the structural unit of open-field behavior in rats [22]. The general level of script is that of the entire session of open-field behavior (the summation of roundtrips over time). Quinpirole-injected rats tested with four objects focused on a few specific places that they visited repeatedly (present data), performing repeatedly and excessively place-specific rituals at these locations [12,15]. Thus, these rats meet the model of Boyer and Lienard [19] in shifting from the level of round trips to repeated visits to a few specific places (simple gestures).

Quinpirole-injected rats that were introduced into the arena with 16 objects typically had only one key location that they visited repeatedly and excessively (Figures 2 and 3). Thus, locomotor rituals in the arena with 16 objects did not undergo the same shift to basic gestures. Rather, these rats kept traveling repeatedly back to the start corner, reducing the variability in their behavior. The stereotyped manner of behavior in these rats was thus a result of an increase in the rigidity of motor performance restricted to a particular route. Such processes were described in amphetamine stereotypy, suggesting that locomotor behavior is organized into a single package as a strategy of coping with drug-induced reduction in information-processing capacity [23]. In that sense, the shift to a single and relatively fixed route is another version of the shift to basic gestures. Rituals in quinpirole-injected rats thus seem to support the hypothesis on ritual formation by shifting focus to a more basic level of organization of motor behavior. This process further supports quinpirole-injected rats as a model of OCD, since a similar shift to basic gestures was shown in motor behavior of OCD patients [21]. Indeed, descriptive tools derived from the quinpirole rat model of OCD are applicable in analyzing, characterizing and treating OCD [6,14].

### Why do quinpirole-treated rats preserve their routes under subsequent environmental changes?

As described above, when exposed to a large arena after 10 preceding exposures to a small arena, both quinpirole and saline-treated rats modified their behavior according to object density and spacing (injections 11–12). A subsequent change in the number of objects and their spacing within the arena without a change in arena size (injections 13–14) did not affect the behavior of the rats established in the previous test phase (injections 11–12). In other words, rats that were introduced into a large arena with four objects kept traveling between 1–2 objects and the start corner even when objects were added. Similarly, a rat traveling in the spaces between objects in a 16-object arena continues to travel in this manner when switched to a four-object arena. Once established, therefore, compulsive-like behavioral patterns in quinpirole-treated rats were anchored in the environment and withstood further changes as long as the anchors (start corner and/or objects) were accessible. This mode of ignoring those environmental changes that do not interfere with ritual performance parallels OCD, where compulsive rituals stem from a failure in inhibitory control [24]. Indeed, in human rituals people feel that they should perform a ritual in the precise way it was performed before, and tend to attach negative emotions to any deviation from that remembered pattern [19]. This was nicely illustrated by Konrad Lorenz [25], describing "*...how tenaciously little children cling to every detail of the accustomed, and how they become quite desperate if a story-teller diverges in the very least from the text of a familiar fairy-tale*". Similarly, the rats clung to the accustomed routes and did not divert unless the environment underwent a major reconstruction that prevented the accustomed performance.

### Object number and inter-object distance: what turns a landmark into a beacon?

When placed in a large arena with objects that had been moved further apart from each other, rats typically moved between 1–2 objects, also typically walking over them, in contrast to walking in the spaces between objects in an arena with many adjacent objects. We suggest that in order to serve as a beacon upon which routes converge, an object has to stand out against the background of landscape, as did the four objects in the large arena. In other words, the same object could act as a beacon or as a landmark according to the contextual background [26]. Since the same object or landmark could be a beacon upon which routes converged or a directional cue with the routes just passing nearby it [27], we suggest that it is not the mere physical structure of the object that accounts for route convergence, but the contextual features, such as landmark spacing and density, that may turn a landmark into a beacon. Altogether, it is the distinctness against the background of the environment [26] that turned a particular landmark into a beacon. Accordingly, four objects stood out against the arena background and served as beacons (positional cues), whereas 16 objects with the same properties formed a relatively homogenous environment in which they served as directional cues, polarizing the arena in relation to the start corner. The question remaining, however, is why one or two specific objects (out of four) were utilized as beacons while other objects were hardly visited? This was probably based on landmarks outside the testing arena ('distal' or panoramic cues), or local olfactory cues deposited by the rats at specific locations.

Overall, in the present study, the routes of rats tested in a large arena with four objects converged upon 1–2 objects, as if the objects were providing accurate positional information. In contrast, the routes of rats that were tested in a large arena with 16 identical objects typically passed between the objects, as if using them as a source of directional information (Figure 3). The differential behavior of rats in a large arena with four objects compared with 16 objects indicates that they extract positional information from a few objects and directional information from many similar objects.

### Proximal (local) cues predominate route shape

The impact of proximal and distal cues is a key subject in exploration and navigation. It is assumed that a distant panorama is more stable, providing a frame of reference within which local landmarks may be placed [28]. This was further supported when hippocampal place cell firing was shown to be in relation to the laboratory frame and not to the maze itself [29]. In the present study, rats were exposed to the manipulation of three types: i) change in the location and number of a free-standing array of objects within the arena; ii) change in arena size; and iii) change in the location of the object and the arena within the room. Of these, objects within the arena are proximal and reachable cues [30], while room setting (doors, windows, lights, etc) are distal, non-reachable global cues. The present results following relocation of an array of objects revealed that the rats were traveling from the start corner to the array of objects wherever the array was placed in the arena. Anchoring routes primarily to the start corner implies that a local proximal cue, a specific and "familiar" corner, is the prime location in the spatial organization of progression. This is in agreement with the finding that rats preserved the initial location [31]. A change in the location of the start corner in reference to room frame (distal cues) did not affect the tendency of rats to anchor their routes to this corner, suggesting that upon introduction to the start corner, rats identify local cues and organize their routes in relation to these proximal cues. The dominance of proximal cues is further supported by the finding that these rats stretched their routes from the start corner to the object array, regardless of the location of the array in relation to arena walls and room frame ('four-wall shift', 'two-wall shift', and 'translocation' tests). This dominance of local cues is in agreement with the finding that rodents use a local view of objects in relation to a stable element of their environment within the open field rather than elements of the distal environment [4,32], thus demonstrating that locomotion of rodents in novel areas is guided by directly localized proximal cues.

### Exploration and navigation in quinpirole compared with saline-treated rats

We used rats treated with quinpirole since these rats are very active and travel repeatedly and incessantly along the same routes with a strong affinity to a few specific locations in the open field [8,11]. Past studies in quinpirole-treated rats [11,12] revealed that the spatiotemporal organization of their behavior is similar to that described in other rodents, including intact rats [33-36]. For example, environmental factors such as objects and corners similarly shape behavior in both quinpirole and saline-treated rats [11,12,15]. Indeed, our results in the first set of experiments with quinpirole resembled those obtained with saline-treated rats, except that the effect of environmental changes was indisputable in quinpirole-treated rats by virtue of their higher activity, compared with a faint effect in saline-treated rats due to their lesser activity. This pattern of results enables extrapolation from the behavior of quinpirole-treated rats to general navigation in freely-moving rats. Unfortunately, by injection 17 on, saline-treated rats had become entirely habituated and ceased to progress in the environment. Consequently, for Experiment 2 we could not verify that the results obtained under quinpirole were also applicable for saline-treated rats.

The comparison of saline and quinpirole-treated rats was based on sampling in each group's peak period of activity [15]. Of the one-hour observation, in saline-injected rats these were the first 15 minutes, and in quinpirole-treated rats these were the last 15 minutes. Sampling the same time interval was not practical since quinpirole-treated rats are inactive immediately after drug administration [9], and their activity gradually increases, peaking at about 40 minutes and on [11]. In contrast, saline-injected rats are most active in the first minutes after being introduced into the open field, and their activity then decreases, so that after 20 minutes they spend most of the time crouching in one place. Sampling the interval of peak activity for each treatment was previously found to properly highlight the difference between quinpirole and saline-treated rats without a meaningless comparison of the behavior in one group with no-behavior of the other group [15].

The use of the same rats in two successive experiments limits the extension of the results of Experiment 2 to saline-treated rats. While quinpirole rats preserved their behavioral pattern over numerous chronic injections (over 40 injections; [11]), in Experiment 2 saline-injected rats were habituated to the large arena to which they had been exposed during Experiment 1. Therefore, further support in saline-treated rats is required for the findings of Experiment 2, that quinpirole-injected rats were traveling from the start corner to the object array regardless of the location of the array in the arena.

### Do rats encode a geometric array of objects or only discrete objects?

An object can be recognized as a unique (separate) object or as a part of a geometric arrangement, but not both simultaneously, depending on the context or the observer's view [27]. Cheng [37] suggested that in navigating and exploring, animals perceive and utilize the overall non-metric geometry of the environment (see [38] for review). For example, a square is perceived as one geometric, non-metric shape, and not as four separate lines. According to the geometric module concept, rats in the present study should similarly perceive the structure of an equispaced square array of four objects in a small (1 × 1 m) or in a large (2 × 2 m) arena, since both comprise the same geometry and differ only in the distance between objects. Perceiving geometric shape was previously demonstrated in hamsters [39] but not in rats [40]; however, the present results support the encoding of a geometric module by rats, since when exposed to 'elastic' expansion of an arena they preserved the same overall preference for certain objects that they had had before arena expansion (Figure 3). The results obtained here follow the prediction of Jacobs and Schenk [27], suggesting that "*a given sketch will be recognized as such in spite of changes in size, although this might induce a general activity response*".

## Conclusion

Rats in the present study appeared to adjust and update their routes according to the new object setting as if they were updating space representation [41]. Readjusting routes to a new object location indicates that the rats were not encoding distances or directions of objects. The degree to which animals respond to spatial changes is probably influenced by variables such as the size of the test environment, the extent of the change relative to the enclosure, and prior experience in the previous setting [32]. The present results support the view that elements in the environment are perceived as a geometric shape, that the same object may exert either positional or directional information according to context, and that an object serves as a beacon only when it stands out against the background of the environment – all described here in the behavior of freely-moving rats. We also show that route shape is primarily dictated by proximal cues within the arena, with priority of the enclosure (walls and corners) over objects that stand alone in the arena. All these facets of exploration and navigation are characteristics of animal cognition [42] as conceptualized by Wise [43]: *"Space is marked and shaped physically with objects forming borders, walls and fences... The marker (wall, road, line boarder, post, sign) is static, dull and cold. But when lived (encountered, manipulated, touched, practised), it radiates a milieu, a field of force, a shape of space"*.

## Methods

### Animals

Following previous studies on chronic quinpirole administration [11], we used male Long Evans Hooded rats. Thirty-three rats at the age of three months and weighing about 200 g were housed in six cages (60 × 40 × 25 cm; 5–6 individuals per cage) in a room with12/12 hours of light/dark and 24°C. Standard rat food, water and fresh sliced apples and carrots were provided ad lib. Each cage included animals from the different test groups, marked by colored ink on the tail. Rats were handled daily for the two weeks before testing.

### Drug

Injection regime and protocol followed past studies [11,12]. Eighteen rats were injected subcutaneously with 0.5 mg/kg (1 ml/kg) of quinpirole hydroclorid (RBI Israel) in the nape of the neck. Drug was injected twice a week, at intervals of 3–4 days. Fifteen control rats were injected with equivolume of saline. Overall, each rat was injected 22 times over 11 weeks.

### Apparatus

The open field was a walled arena with PVC navy-blue floor and 60 cm high opaque walls, placed in a temperature-controlled room (24°C) illuminated with two 300 w light bulbs that were directed to the ceiling in order to provide diffused indirect light. The room panorama that was viewable for the rats included numerous objects such as door, window, shelves, enabling the animals to locate their location within the arena in reference to the room layout. A video camera (Ikeami B/W ICD-47E) was mounted above the arena, providing a top view that was continuously recorded onto a VCR (JVC HR-J737). According to the experimental design detailed below, arena size was adjusted to 1 × 1 m (small arena) or 2 × 2 m (large arena). Cement bricks (11 × 11 × 8 cm each), painted in smooth blue paint served as 'objects' and were placed in the open field as detailed below for each experiment.

### Procedure

Rats first underwent eight sessions (two per week over four weeks). In each session, an individual rat was injected with quinpirole, and immediately after drug administration placed at the near left corner of the 1 × 1 m arena, facing the center of the arena. Behavior was then videotaped for 60 minutes. Four objects were placed in the arena, spaced at equal distances from the walls and from each other. After the 60 min session, the rat was returned to its home cage and the arena was cloth-wiped with detergent. All sessions took place during daytime (8 am-6 pm).

### Data acquisition and analysis

Videotapes of injections 9–22 were analyzed by means of *'Ethovision' *(by Noldus, NL) software. This computer program tracks the progression of the rat in the arena, providing the time and the location of the center of the head of the animal five times per second. Following the analyses of past studies of quinpirole-treated rats and their respective saline-treated controls, behavior was analyzed only at the time of peak activity, which was between 40–55 min in quinpirole-treated rats, and between 0–15 min in saline-injected rats [15]. The rationale for the selected time intervals was to compare the periods of peak activity in saline and quinpirole-treated animals. Indeed, quinpirole-treated rats are least active immediately after drug administration [8], when saline-treated rats are most active. However, behavioral peak in quinpirole-treated rats also depends on the exposure to the test environment [11]; thus, rats could not be injected elsewhere and introduced into the test arena 40 minutes later, but had to be introduced into the arena immediately after drug injection. In contrast, saline-treated rats are entirely habituated 40–55 min after being introduced to the open field, when quinpirole-treated rats are at their peak activity. *Ethovison *data were then transferred to *Microsoft Excel*, and filtered to bouts of progression and stopping, where a stop was defined as not progressing for at least 0.4 sec. Filtration was required due to the measuring method of *Ethovision*, which is based on identifying the rat's surface area and giving the x-y coordinates of its center. However, if a rat was active during stopping (e.g., rearing on its hindquarters, grooming etc.), its surface area changed, and this change was identified by *Ethovision *as progression, although the rat was actually stopping (noise data). To filter out this noise, we first observed some of the videotapes, looking for distinct progress and stop bouts. Noting the start and end time and traveling distance of these intervals, we found that the minimum stopping period was 0.4 sec in which the rat was not progressing. After filtration, we calculated two parameters: 1. *Traveled distance*: overall metric distance that a rat traveled during the analyzed 15-min; 2. *Interstop distance*: metric distance traveled between two consecutive stops. It should be noted that other parameters that characterize open-field behavior were extracted from *Ethovision*, for example, traveling speed, number of stops, number of interstop segments, etc. However, differences in these variables mainly reflected the well-documented higher activity in quinpirole rats, and therefore these variables are not shown in the Results section.

### Statistics

Starting with injection 9, rats were tested at each two successive injections under a specific setting of objects. In order to reduce variability stemming from anecdotal behavior of an individual rat (for example, staying for an extended period at one location for coprophagia or licking), we averaged the behavior of each individual rat over the two successive sessions with the same setting. For each of the above parameters, we then performed a two-way ANOVA in order to reveal the following differences: i) between quinpirole and saline-treated rats (between-group factor); ii) between object settings in each treatment group (within-group factor); and iii) the interaction of treatment and setting. Since the above parameters were not entirely independent, p level underwent Bonferroni adjustment to 0.01. Significance in the two-way ANOVA was followed by a Tukey HSD test for unequal samples.

## Authors' contributions

PZ and DE contributed equally to this study, which is partially based on M.Sc. study of SR. All authors read and approved the final manuscript.
